# Sorghum CCoAOMT and CCoAOMT-like gene evolution, structure, expression and the role of conserved amino acids in protein activity

**DOI:** 10.1007/s00438-018-1441-6

**Published:** 2018-05-02

**Authors:** M. Rakoczy, I. Femiak, M. Alejska, M. Figlerowicz, J. Podkowinski

**Affiliations:** Institute of Bioorganic Chemistry PAS, ul. Noskowskiego 12/14, 61-704 Poznan, Poland

**Keywords:** Sorghum (*Sorghum bicolor* L.), CCoAOMT, Gene expression, Phylogeny, Protein structure

## Abstract

Sorghum is a crop plant that is grown for seeds, sucrose, forage and biofuel production. In all these applications, lignin is a superfluous component that decreases the efficiency of technological processes. Caffeoyl-coenzyme A *O*-methyltransferase (CCoAOMT) is an enzyme involved in monolignol synthesis that affects the efficiency of lignification and lignin composition. The sorghum genome harbors one CCoAOMT gene and six closely related CCoAOMT-like genes. The structures of four sorghum CCoAOMT-like enzymes suggest that these proteins might methylate caffeoyl coenzyme A and contribute to monolignol synthesis. In this study, two sorghum genes, CCoAOMT and one CCoAOMT-like, were found to be highly expressed in leaves, stems and immature seeds. The promoters of these genes possess clusters of transcription factor-binding sites specific for lignification, and this suggests that they are important for lignification. Phylogenetic analysis revealed that one sorghum CCoAOMT-like enzyme is closely related to ancestral cyanobacterial CCoAOMT-like proteins. The remaining CCoAOMT-like enzymes, including the one highly expressed in the leaves and stem, are closely related to CCoAOMT. Genes from these two groups possess different, evolutionarily conserved gene structures. The structure of the sorghum CCoAOMT-like protein from the ancestral clade was modeled and differences between enzymes from the two clades were analyzed. These results facilitate a better understanding of the evolution of genes involved in lignification, and provide valuable data for sorghum improvement through traditional breeding or molecular genetic techniques. The findings suggest that CCoAOMT-like genes might be recruited in lignification and raise questions of the frequency of such functional shifts.

**Electronic supplementary material:**

The online version of this article (10.1007/s00438-018-1441-6) contains supplementary material, which is available to authorized users.

## Introduction

Sorghum (*Sorghum bicolor* L., 2*n* = 20), which belongs to the Poaceae family, is a C4 crop plant that is cultivated for seeds, sugar and green biomass used as forage (Carpita and McCann 2008; Mohammed et al. 2015). Recently, sorghum was used for biofuel production, and it is a model bioenergy plant (Calvino and Messing 2012; Mullet et al. 2014; Vermerris 2011). In all these applications, lignin—the second most abundant component of the plant cell wall—positively affects plant growth and biomass production, but it decreases the efficiency of technological processes. Attempts to improve the quality of plant material drew attention to caffeoyl-coenzyme A *O*-methyltransferase (CCoAOMT), one of the enzymes involved in lignin monomer synthesis (Anterola and Lewis 2002; Bottcher et al. 2013; Goujon et al. 2003). The sorghum genome harbors one CCoAOMT gene and six closely related genes coding for CCoAOMT-like enzymes (Walker et al. 2016; Xu et al. 2009). To investigate the impact of these genes on lignification, it is necessary to explore their expression pattern, association with biological processes and phylogenetic relationships.

CCoAOMTs and CCoAOMT-like enzymes form a family within *O*-methyltransferases characterized by molecular weight ranging between 26 and 29 kDa and divalent metal ions in the active site (Ferrer et al. 2005; Joshi and Chiang 1998; Lee et al. 2008; Liu et al. 2016; Walker et al. 2016; Widiez et al. 2011). Despite the similarities, CCoAOMTs are substrate specific and use two methyl acceptors, caffeoyl-coenzyme A or 5-hydroxyferuloyl-coenzyme A, but CCoAOMT-like enzymes use a variety of compounds, including flavonoids, anthocyanins, phenylpropanoids or alkaloids (Ibdah et al. 2003; Inoue et al. 1998; Widiez et al. 2011). The enzymes from these two groups are usually associated with different biological processes. CCoAOMTs provide precursors for coniferyl and sinapyl alcohols, which are subsequently used in lignin synthesis (Barros et al. 2015; Dixon et al. 2001; Raes et al. 2003; Vanholme et al. 2010), and CCoAOMT-like enzymes, which process a broad spectrum of substrates, are involved in a variety of secondary metabolism pathways that are often specific for a given plant. The role of CCoAOMT in lignin synthesis was first recognized in *Z. violacea* and *N. tabacum*, where the enzyme was identified in all lignified tissues (Maury et al. 1999; Ye et al. 1994; Zhong et al. 1998). The direct evidence of CCoAOMT’s role in lignin synthesis came from transgenic plants with increased or decreased CCoAOMT gene expression (Guo et al. 2001; Ye et al. 2001). Different approaches used to downregulate CCoAOMT, including antisense RNA, RNA interference, or gene knockout, resulted in decreased lignin content in plants, which was associated with collapsed vascular bundles, lower height, lower biomass and developmental retardation (Do et al. 2007). Plants with downregulated CCoAOMT genes also exhibit modified lignin subunit composition due to disturbances in syringyl and guaiacyl monolignol synthesis (Li et al. 2013; Meyermans et al. 2000; Pang et al. 2014; Pinçon et al. 2001; Toraman et al. 2016; Zhong et al. 1998). CCoAOMT overexpression produces the opposite effects, including increased lignin content, plant height and silique length (Zhang et al. 2014).

Recent results suggest that the role of substrate-specific CCoAOMTs is not limited to lignin synthesis and secondary cell wall formation. The CCoAOMT from petunia was shown to participate in the phenylpropene pathway leading to the synthesis of volatile attractants in petals (Shaipulah et al. 2016). The *Vitis vinifera* substrate-specific CCoAOMT is a multifunctional *O*-methyltransferase that may contribute to anthocyanin methylation (Giordano et al. 2016). Maize ZmCCoAOMT2, aside from its role in lignin synthesis as an enzyme, was shown to confer quantitative resistance to fungal pathogens (Wang and Balint-Kurti 2016; Yang et al. 2017). The process depends on ZmCCoAOMT2 interactions with nucleotide-binding leucine-rich-repeat proteins and suppression of a hypersensitive response.

Several lines of evidence demonstrated that CCoAOMT-like enzymes might contribute to monolignol synthesis. Most CCoAOMT-like enzymes are usually associated with species-specific processes (Ibdah et al. 2003; Kopycki et al. 2008a; Widiez et al. 2011), but the sorghum enzymes from this group were suggested to methylate the same substrate as substrate-specific CCoAOMTs (Walker et al. 2016). The crystal structure comparison of the substrate-specific CCoAOMTs from the dicot plant *Medicago sativa* and the monocot plant *S. bicolor* with the promiscuous CCoAOMT-like enzyme from *M. crystallinum* revealed that differences in substrate specificity result from small topological changes in the N-terminal amino acids and a C-terminal variable loop (Ferrer et al. 2005; Hoffmann et al. 2001; Kopycki et al. 2008a; Walker et al. 2016).

The evolutionary relationships between CCoAOMT and CCoAOMT-like enzymes are more complex than classification based on substrate specificity. CCoAOMT-like enzymes are divided into two clades: one grouping CCoAOMT-like enzymes closely related to ancestral, cyanobacterial CCoAOMT-like and another, which comprises close relatives of substrate-specific CCoAOMTs (Ibdah et al. 2003; Ma and Luo 2015; Sahr et al. 2010; Widiez et al. 2011). All sorghum CCoAOMT-like enzymes for which the previous structural analysis suggests CCoAOMT activity are closely related to substrate-specific CCoAOMTs (Walker et al. 2016). However, the importance of these enzymes for lignification processes depends also on efficient expression in proper tissues.

In this study, the expression of sorghum CCoAOMT and CCoAOMT-like genes was examined in highly lignified organs: mature leaves, stem and immature seeds. Two genes were found to be highly expressed in these organs: substrate-specific CCoAOMT and one of the CCoAOMT-like genes. The promoters of these genes possess clusters of transcription factor-binding sites that are specific for lignification, suggesting a role in lignification processes. The phylogenetic analysis of sorghum enzymes and well-characterized homologous proteins from plants and cyanobacteria revealed early dedifferentiation of CCoAOMTs and CCoAOMT-like enzymes, from CCoAOMT-like enzymes related to ancestral cyanobacterial enzymes. These two clades differ in terms of exon–intron organization of their genes. The amino acids essential for CCoAOMT and CCoAOMT-like enzymes were identified using protein structural analysis, homology modeling and conservation analysis. These data suggest that sorghum CCoAOMT-like genes might be recruited to monolignol synthesis and lignification processes and shed new insights into the origin of substrate-specific CCoAOMTs. The results concerning CCoAOMT and CCoAOMT-like genes are associated with long-distance transport in plants, plant response to pathogens, mechanical resistance, and the quality of plant material used as food or forage, and may be applied to improve sorghum through traditional or molecular breeding.

## Materials and methods

### Plant materials

*Sorghum bicolor* cv. RONA1 plants grown in the field for 4 months (110 days) were in the growth stage III, between flowering and grain formation, when the plant material was collected (Gerik et al. 2003). The plant samples, consisting of mature leaves, stems and immature seeds from up to five plants, were pooled and flash frozen in liquid nitrogen a few minutes after collecting.

### RNA isolation

Total RNA was isolated from mature leaves, the inner section of the stem, or immature seeds, frozen in liquid nitrogen and ground into powder in a mortar in the presence of liquid nitrogen. RNA was isolated with RNeasy Plant Kit (Qiagen) or extracted with 0.1 M NaCl, 2% SDS, 50 mM Tris/HCl pH 8.0, 10 mM EDTA buffer in the presence of phenol–chloroform–isoamyl alcohol and precipitated two times with 2 M LiCl, resuspended in RNase-free water and precipitated with 2.5 volumes of ethanol. Finally, RNA was resuspended in RNase-free water. RNA concentration and quality was evaluated using UV absorbance at 260 nm followed by electrophoresis on an agarose gel under denaturing conditions (Sambrook et al. 1989) or RNA 6000 Nano Chip (Agilent). The reverse transcription (RT) reaction was carried out using Maxima First Strand cDNA Synthesis Kit for RT-qPCR (Thermo Scientific) with 100–500 ng/µL of total RNA as a template.

### Digital droplet PCR (ddPCR)

Reverse transcription (RT) was carried out using Maxima First Strand cDNA Synthesis Kit for RT-qPCR (Thermo Scientific) with 100–500 ng/µL of total RNA as a template. A single ddPCR reaction was composed of 1.0 µL of cDNA samples (RT reaction) diluted 1:5–1:50 in water, 200 nM primers (Table 1), QX200 ddPCR EvaGreen Supermix (Bio-Rad) and MilliQ water to a total reaction volume of 21 µL. The reaction was partitioned into droplets by the QX200 Droplet Generator (Bio-Rad) and amplified in a C1000 Touch Thermal Cycler (Bio-Rad). The amplification conditions were composed of initial denaturation at 95 °C for 5 min, 40 cycles of 95 °C for 30 s, 58 °C for 30 s and 72 °C for 45 s, and a final extension at 72 °C for 2 min followed by signal stabilization at 4 °C for 5 min and 90 °C for 5 min. After completing the amplification, the fluorescence of droplets was registered using a QX100 droplet reader (BioRad) and data were analyzed using QuantaSoft software. The expression of *S. bicolor* genes was normalized using ubiquitin carrier protein-Sobic.009G174900 (Johnson et al. 2014).

**Table 1:** List of primers

Application		ID	Primer sequence, from 5′ to 3′
*S. bicolor* genes RT-ddPCR expression analysis	SbCCoAOMT-2	Mz47	TTCGTGGACGCGGACAAGGT
		Mz48	ATGGCGGCGTTGAACTCCCT
	SbCCoAOMT-6	Mz49	AAGGTGGAGTTCCGAGGGCA
		Mz51	CAGCGTCGACGAACACGAAGT
	SbCCoAOMT-5	Mz52	AACCTCGGCGCCTTCGACTT
		Mz53	GCGGCAATCACGGCGTTGAA
	SbCCoAOMT-4	Mz56	GCGATCAAGGACCTCAATGT
		Mz57	TGAGTGATGAGTTCCGACGA
	SbCCoAOMT-3	Mz62	GGTGGCAGCATCCCGAATGT
		Mz63	TGTTCCTCCGCTCGTGCTTG
	SbCCoAOMT-7	Mz86	GGGCTTCCTCGGTAGATTTC
		Mz87	GGCGATGAACCTCTTGACAT
	SbCCoAOMT-1	S95	TTCTTGGGAATTGTCGCCAT
		S96	ATCCACGGTAGGAGCAGTAT
	Sobic.009g174900	S99	CTGACGATTACCTTCTGCTC
		S100	CCTCACTATGGATGGCAATG

### Bioinformatic and phylogenetic analysis

Sequences were assembled, edited and processed with BioEdit software, BLAST and The Sequence Manipulation Suite (Stothard 2000).

ChloroP 1.1 was used to predict chloroplast transit peptide in protein sequences (Emanuelsson et al. 1999) and CELLO2GO was used for protein subcellular localization (Yu et al. 2014).

The CCoAOMT amino acid sequences were aligned using ClustalW. A phylogenetic tree was constructed using MEGA 6.0 software (Tamura et al. 2013) with the maximum likelihood method based on the JTT matrix-based model (Jones et al. 1992). Bootstrap-supported consensus trees were inferred from 1000 replicates (Felsenstein 1985) and branches with less than 40% bootstrap support were collapsed.

UCSF Chimera, a visualization system for exploratory research and analysis, was used for structure conservation analysis.

Conservation mapping of the corresponding sequence alignment onto the structure of SbCCoAOMT-1 (PBD:5KVA) was performed using Chimera with the default setting. For conservation style, AL2CO was chosen with sum of pairs as the type of equation.

The 3D model of the SbCCoAOMT-7-trimmed protein was predicted to examine structural differences between the CCoAOMT-like protein from clade 2 and SbCCoAOMT-1 (PDB: 5KVA) from clade 1a. Structure modeling was performed using the I-TASSER server with default structural template selection. I-TASSER (Iterative Threading ASSEmbly Refinement)—http://zhanglab.ccmb.med.umich.edu/I-TASSER.

Databases:

Phytozome (https://phytozome.jgi.doe.gov),

GenBank (https://www.ncbi.nlm.nih.gov/genbank/),

PLACE (Higo et al. 1997) (http://bioinformatics.psb.ugent.be/webtools/plantcare/html/).

Figures and diagrams presenting genes and promoter regions structure were prepared with Illustrator for Biological Sequences (IBS) (Liu et al. 2015) and Gene Structure Display Server (GSDS) (Hu et al. 2014).

### Primers used in this study

See Table 1.

### Accession numbers of sequences used in this article

Sequence data from this article can be found in the Phytozome and GenBank/EMBL data libraries, ID of genomic sequences used for gene structure analysis, which are not linked to protein sequences are given in parenthesis: *Arabidopsis thaliana*—AT4G34050.1, AT1G24735.1, AT1G67980, AT1G67990, AT4G26220.1, AT3G62000.1, AT3G61990.1; *Chlamydomonas reinhardtii*—XP_001693484 (gene Cre13.g571950.t1.2); *Medicago truncatula*—Medtr4g094815, Medtr4g085590, Medtr4g094368, Medtr2g070410, *Medicago sativa*—AAC28973; *Mesembryanthemum crystallinum*—AAN61072; *Nicotiana tabacum*—AAC49913, CAA91228, CAB05369; *Oryza sativa*—BAA78733 (gene LOC_Os06g06980.1), AAT68023 (gene LOC_Os08g38900.1), BAD08718 (gene LOC_Os08g38910.1), BAG98414 (gene LOC_Os09g30360.1), *Petroselinum crispum*—AAA33851; *Physcomitrella patens*—Pp3c23_5530V3.1, Pp3c4_20870V3.5; *Populus—*BAA19102; *Plagiochasma appendiculatum*—ALS88170; *Selaginella moellendorffii*—locus 101568, XP 002963752 (gene NW_003314264 REGION: 2755091..2756705), XP002976256 (gene NW_003314291 REGION: 568356..569800); *Sorghum bicolor*—Sobic.010G052200.1 (SbCCoAOMT-1), Sobic.002G242300.1 (SbCCoAOMT-2), Sobic.007G218700.1 (SbCCoAOMT-3), Sobic.007G218800.1 (SbCCoAOMT-4), Sobic.007G218500.1 (SbCCoAOMT-5), Sobic.007G217200.1 (SbCCoAOMT-6); Sobic.007G043200.1 (SbCCoAOMT-7); Sobic.007G047300.1 (COMT, caffeic acid *O*-methyltransferase), Sobic.009G174900 (ubiquitin carrier protein); *Synechocystis* sp.—WP_010873795.1; *Vanilla planifolia*—ADZ76153, ADZ76154; *Zea mays*- CAB45149 (gene GRMZM2G127948), AFW65160 (gene GRMZM2G332522_T02), DAA40360 (gene GRMZM2G004138_T01), XP_008662866 (gene GRMZM2G077486_T01).

## Results

### *S. bicolor* genome harbors seven gene coding for CCoAOMT and CCoAOMT-like proteins

The *Sorghum bicolor* genome was queried with sequences of well-characterized caffeoyl-coenzyme A *O*-methyltransferases (CCoAOMT) and CCoAOMT-like proteins. This yielded seven genes: Sobic.010G052200, Sobic.002G242300.1, Sobic.007G218700, Sobic.007G218800.1, Sobic.007G218500, Sobic.007G217200 and Sobic.007G043200, Table 2. In this study, the genes and their peptides were renamed SbCCoAOMT-1, SbCCoAOMT-2, SbCCoAOMT-3, SbCCoAOMT-4, SbCCoAOMT-5, SbCCoAOMT-6 and SbCCoAOMT-7, Table 2. Screening the sorghum genome and sequence databases with these sequences identified genes or cDNAs coding for the same seven proteins or more distantly related sequences, such as Sb07g00386, of higher similarity to COMT than CCoAOMT or CCoAOMT-like proteins.

**Table 2:** *S. bicolor* CCoAOMT and CCoAOMT-like protein names used in this study and physicochemical parameters: MW—molecular weight, pI—isoelectric point, GRAVY—the grand average of hydropathy

Gene/protein name in this study	Locus id in phytozome	Protein length	MW (kDa)	pI (pH)	GRAVY [− 2 .. + 2]	Predicted subcellular localization	Clade
SbCCoAOMT-1	Sobic.010G052200.1	261 aa	29.07	5.05	− 0.261	Cytoplasm	1a
SbCCoAOMT-2	Sobic.002G242300.1	241 aa	25.73	5.08	0.139	Chloroplast	1c
SbCCoAOMT-3	Sobic.007G218700.1	250 aa	27.36	5.35	− 0.155	Cytoplasm, Chloroplast	1c
SbCCoAOMT-4	Sobic.007G218800.1	246 aa	27.23	4.87	− 0.107	Cytoplasm	1c
SbCCoAOMT-5	Sobic.007G218500.1	267 aa	28.76	5.34	0.005	Cytoplasm, chloroplast	1c
SbCCoAOMT-6	Sobic.007G217200.1	144 aa	15.91	4.38	0.041	Cytoplasm	1c
SbCCoAOMT-7	Sobic.007G043200.1	306 aa	32.99	9.06	0.103	Chloroplast	2
SbCCoAOMT-7 trimmed at Ser-67		239 aa	26.37	7.30	0.016		2

Protein subcellular localization was analyzed with CELLO2GO. The trimmed SbCCoAOMT-7 transit peptide at Ser-67 is based on ChloroP analysis

**Fig. 1:**
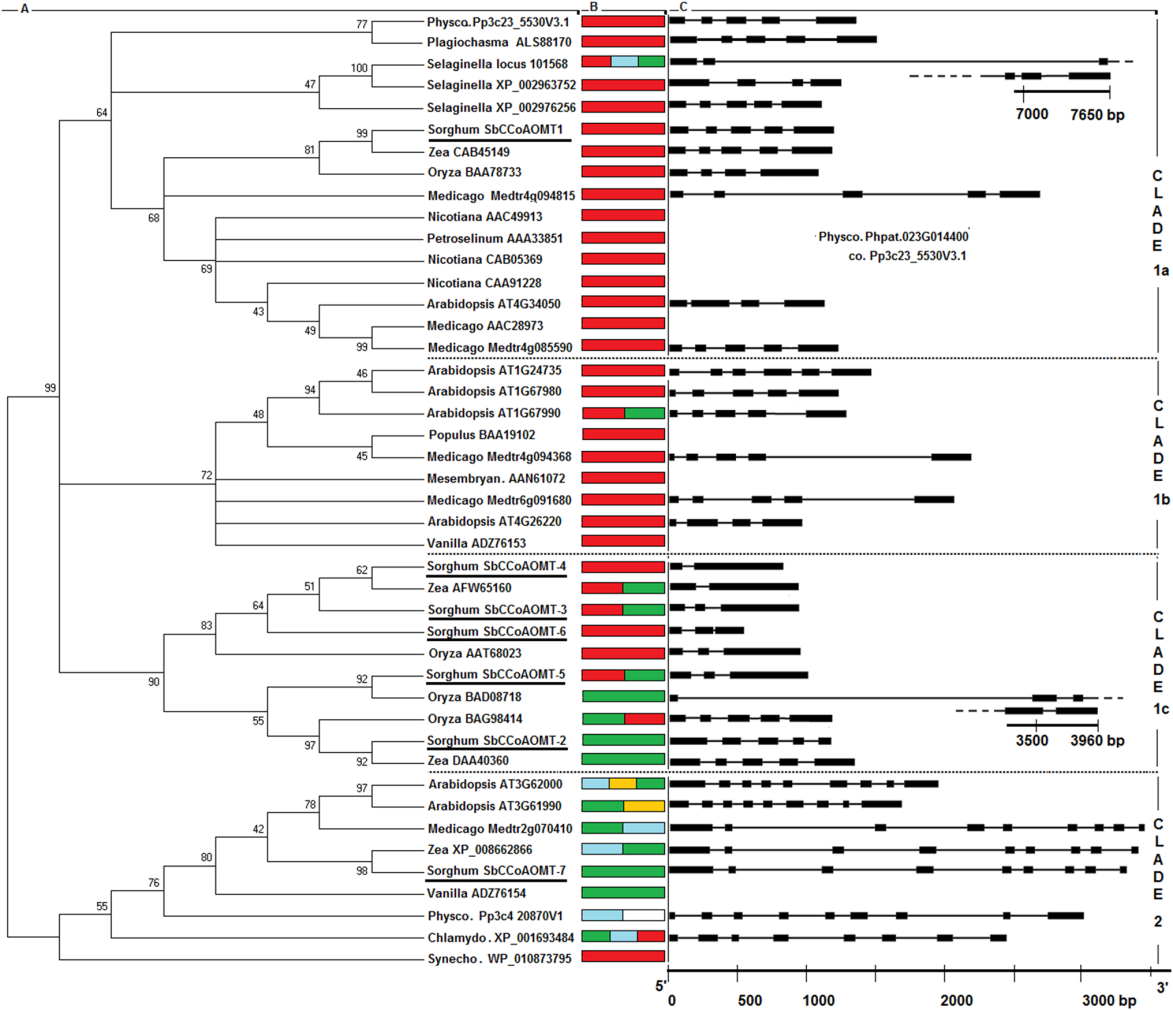
Phylogeny, subcellular localization and gene structure of plant CCoAOMT and CCoAOMT-like proteins. The analyzed proteins include the sorghum proteins (underlined) and their well-characterized homologs. A—the unrooted, maximum likelihood tree was constructed with 1000 bootstrap replications; the original tree was condensed with a 40% cut-off value. The clades are named according to Widiez et al. (2011): clade 1a—true CCoAOMTs; clade 1b—non-grass CCoAOMT-like enzymes; clade 1c—grass CCoAOMT-like enzymes; and clade 2—CCoAOMT-like proteins closely related to cyanobacteria. The peptide IDs or accession numbers are proceeded by genus name or shortcut: Physco.—*Physcomitrella*, Chlamydo.—*Chlamydomonas*, Synecho.—*Synechocystis*; B—subcellular localization identified with CELLO2GO web server: red—cytoplasmic, green—chloroplast, blue—mitochondrial, yellow—nuclear, white—extracellular; C—the gene structure within the coding region with exons marked by black boxes and introns by lines. (Color figure online)

The evolutionary relationships among the sorghum CCoAOMT and CCoAOMT-like proteins and their homologs from other plants were ascertained using Mega 6, Fig. 1. The sorghum CCoAOMT and CCoAOMT-like genes were categorized into three clades that were named according to Widiez et al. (2011): clade 1a—true CCoAOMTs; clade 1c—grass CCoAOMT-like proteins; and clade 2—CCoAOMT-like proteins closely related to cyanobacterial enzymes, Fig. 1. SbCCoAOMT-1 is the only sorghum protein in clade 1a, the grouping of CCoAOMT enzymes with high substrate specificity. This clade harbors CCoAOMTs associated with lignin synthesis, including *A. thaliana* ATG434050, *N. tabacum* CAA91228, *Petroselinum crispum* AAA33851, and *M. sativa* AAC28973 (Do et al. 2007; Ferrer et al. 2005; Martz et al. 1998; Pakusch et al. 1989). Five closely related sorghum CCoAOMT-like genes: SbCCoAOMT-2, SbCCoAOMT-3, SbCCoAOMT-4, SbCCoAOMT-5, and SbCCoAOMT-6 are grouped in clade 1c specific for grass CCoAOMT-like enzymes promiscuous with respect to the methyl acceptor. The SbCCoAOMT-7 gene is the only sorghum gene in clade 2, which comprises CCoAOMT-like proteins closely related to cyanobacterial enzymes. Similar to other plant proteins from this clade, the SbCCoAOMT-7 protein has an N-terminal extension of 67 amino acids.

The analysis of CCoAOMT and CCoAOMT-like gene structures supports the phylogenetic relationships inferred from the protein phylogenies. The genes exhibit two different exon–intron patterns within the coding region. Genes composed of five exons are characteristic for superclade 1, but clade 2 genes are composed of nine exons, Fig. 1. Exon boundaries are conserved within clades and differ between superclade 1 and clade 2 even when the exon–intron junctions are localized in conserved regions, Fig. S-1. The clade-specific gene structure is masked in some genes, such as SbCCoAOMT-3, SbCCoAOMT-4 and SbCCoAOMT-5, by 3′ end-terminal intron elimination.

SbCCoAOMT-6, the shortest sorghum CCoAOMT-like gene, possesses a unique exon–intron structure, with exon boundaries different from other genes, Fig. S-1. The location of the SbCCoAOMT-6 exon boundaries is associated with two indels specific for this gene: a duplication of the motif EHLDALLADEG in the central part of the protein and a 30 amino acid deletion in the variable loop, Fig. S-1.

### *S. bicolor* CCoAOMT and CCoAOMT-like proteins: amino acid conservation, structure of superclade 1 and clade 2 proteins

The sorghum CCoAOMT and CCoAOMT-like genes encode proteins 144–306 amino acid residues in length with calculated molecular weights of 15.91–32.99 kDa, Table 2. The shortest protein is SbCCoAOMT-6, which possesses an N-terminal deletion of 90 amino acids, a 34 amino acid deletion in the variable loop and a 16 amino acid insertion in the central region, Fig. S-1. These large deletions suggest that SbCCoAOMT-6 is not a full-length protein, and it is shorter than the cyanobacterial protein WP 010873795. The deletions in the insertion loop of SbCCoAOMT-2 and SbCCoAOMT-7 proteins are shorter, 8–9 amino acids in length, and the length of their insertion loops is similar to *Z. mays* (AFW 65160, XP 008662866), *O. sativa* (AAT 68023) or *A. thaliana* (AT3G62000), Fig. S-1. The longest sorghum CCoAOMT-like protein is SbCCoAOMT-7 due to its highly hydrophobic N-terminal extension. The ChloroP program assigned the first 67 N-terminal amino acids of SbCCoAOMT-7 as a chloroplast transit peptide. A similar transit peptide targeting the enzyme to plastids is present in Vp-OMT5 (ADZ76154) from *Vanilla planifolia*, and AT3G61990 from *A. thaliana* possesses a transit peptide that targets the enzyme to the endoplasmic reticulum (Sahr et al. 2010; Widiez et al. 2011). The apparent N-terminal extension is also present in other clade 1c proteins, and analysis of their subcellular localization with CELLO2GO and ChloroP suggests that SbCCoAOMT-2 is targeted to plastids, while SbCCoAOMT-3 and SbCCoAOMT-5 are targeted to two compartments: cytosol and plastids, Fig. 1 (Yu et al. 2014). Similar to sorghum, *O. sativa* and *Z. mays* clade 1c proteins also possess transit peptides targeting them to plastids, Fig. 1b.

Forty-five highly conserved amino acids preserved across all clades in more than 95% of the analyzed sequences were identified in the set of 43 CCoAOMT and CCoAOMT-like proteins used for the phylogenetic study (SbCCoAOMT-6 was excluded from the conservancy analysis), Fig. S-1 and CCoAOMT-CCoAOMT-like alignment in supplementary files. These amino acids are conserved in the sorghum CCoAOMT and CCoAOMT-like enzymes except SbCCoAOMT-6, which is missing six conserved amino acids due to two large deletions.

**Fig. 2:**
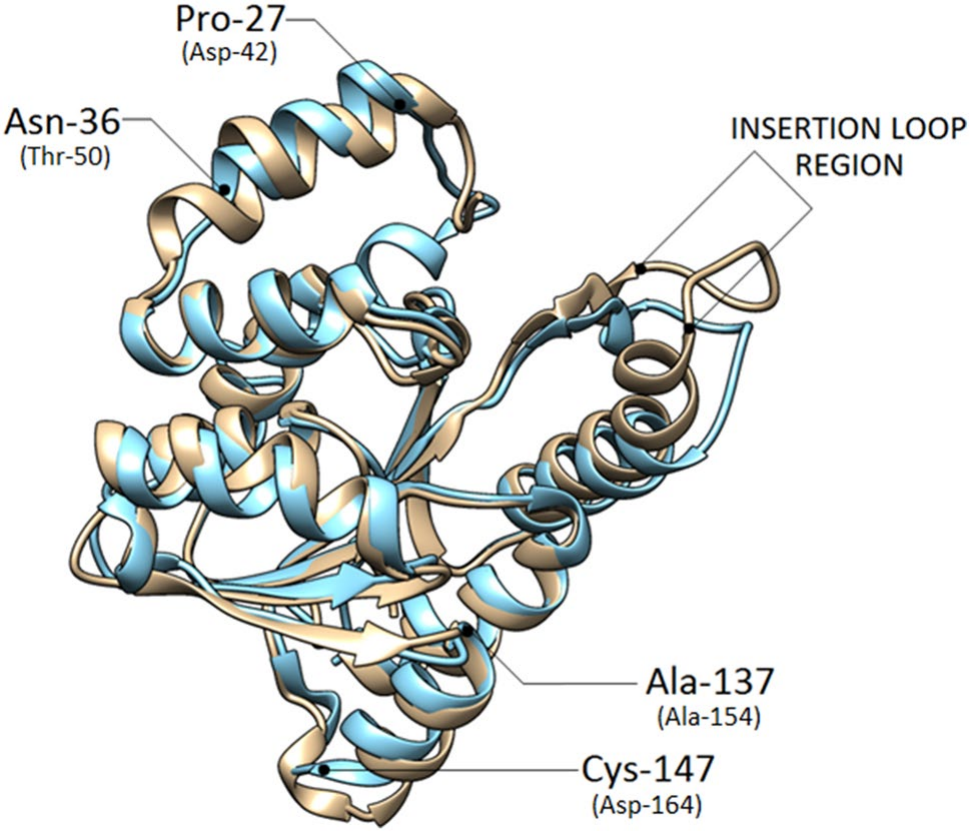
Superimposition of the model of SbCCoAOMT-7 trimmed protein (blue) and the structure of SbCCoAOMT-1, PBD ID: 5KVA (tan). The regions of the highest discrepancy between SbCCoAOMT-1 and SbCCoAOMT-7 are shown: α-helix Pro-27 to Asn-36, α-helix Ala-137 to Cys-147 and variable loop; amino acid coordinates are according to the trimmed SbCCoAOMT-7 protein. The corresponding amino acids of the SbCCoAOMT-1 protein are marked in brackets. (Color figure online)

To investigate structural differences between the substrate-specific enzyme SbCCoAOMT-1 from superclade 1 and CCoAOMT-like protein from clade 2, we compared the crystallographic structure of SbCCoAOMT-1 (PDB ID: 5KVA) with the model of the SbCCoAOMT-7 protein. For structure modeling, the SbCCoAOMT-7 protein was trimmed at Ser-67 to cut off the chloroplast transit peptide absent in the mature enzyme. The structure of the trimmed peptide was predicted using the I-TASSER server, which employs PDB datasets in the structure-threading method. The template structures applied for structure modeling involved two bacterial *O*-methyltransferases structures, PDB ID: 3R3H and 2HNKA, and human catechol-*O*-methyltransferase structure PDB ID: 2AVD, all with normalized *Z* score of above 1 (2.93, 3.97 and 2.81, respectively). Subsequently, the resulting model of the SbCCoAOMT-7 protein with the highest confidence score (*C* score = − 0.08) and the SbCCoAOMT-1 structure (PDB ID: 5KVA) was aligned using MatchMaker in Chimera (Fig. 2). The most divergent region is Asp-195 to Asp-200 of the insertion loop in trimmed SbCCoAOMT-7 protein. The variability of this region is associated with discrimination between different methyl group acceptors. The differences between the SbCCoAOMT-1 and SbCCoAOMT-7 structures also involve the shortening of two α-helices: region Pro-27 to Asn-36 of trimmed SbCCoAOMT-7 (Asp-42 to Thr-50 of SbCCoAOMT-1) and region Ala-137 to Cys-147 of trimmed SbCCoAOMT-7 (Ala-154 to Asp-164 of SbCCoAOMT-1). The first of the α-helices corresponds to the region of reference structure 5KVA involved in dimerization. Furthermore, the other discrepancy between these two structures concerns the N-terminal α-helix localized between Ala-1 and Arg-18 of the trimmed SbCCoAOMT-7, which spatial orientation is ambiguous as this region does not possess any templates in the PDB database.

### Promoter regions of two sorghum CCoAOMT-like genes are enriched in binding sites for transcription factors involved in lignification

**Fig. 3:**
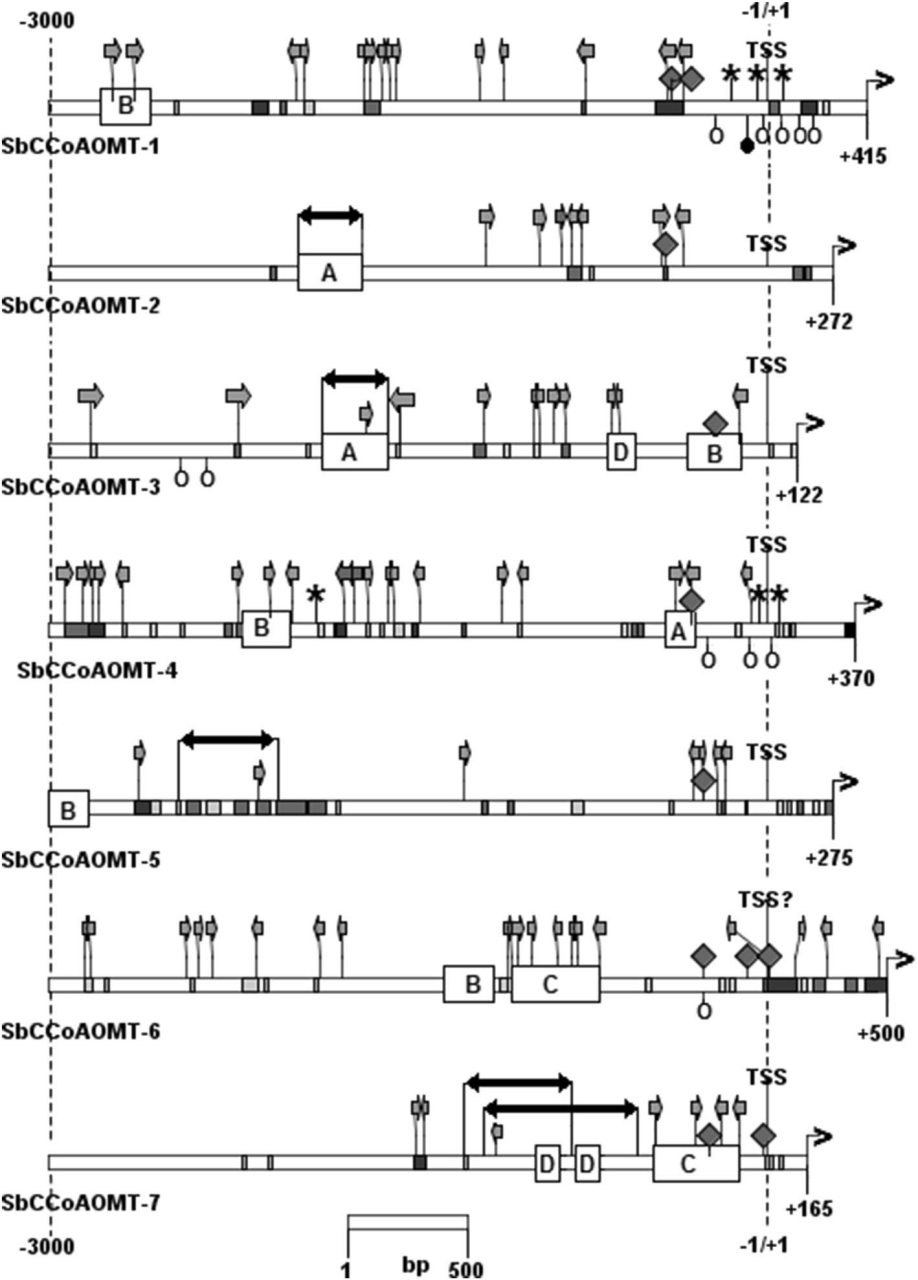
The structure of *S. bicolor* CCoAOMT and CCoAOMT-like promoter regions. The regions comprising 5′ UTR and 3 kb above the transcription start site were screened for inverted and direct repeats—marked with black and gray arrows; short conserved regions—marked as small, gray rectangles; conserved regions longer than 30 bp—marked with big rectangles and labeled A to D; binding sites for transcription factor involved in lignification processes—open circles (MybPlant motif) and black circles (ACIIPVPAL2); motifs similar to AC elements—asterisks; TATA boxes—squares. Transcription start site is marked as TSS and start codon is marked with a black arrow

The promoter region of sorghum CCoAOMT and CCoAOMT-like genes spanning the 5′ UTR and 3 kb above the transcription start site were scrutinized for inverted and direct repeats, conserved regions longer than 15 bp, and binding sites for transcription factors involved in lignification, Fig. 3. The distribution of these elements revealed a high variance in the architecture of the upstream regions of sorghum CCoAOMT and CCoAOMT-like genes. The sequence conservation is weak even within the vicinity of the transcription start sites and 5′ UTRs. Four classes of motifs conserved in at least two of the promoters with 97–74% identity and of 38–361 bp in length were identified and named motifs A, B, C and D, Table S-1. The highest conservation was observed for the 361 bp long motif C present in the SbCCoAOMT-6 and SbCCoAOMT-7 promoter regions. The motifs are not limited to CCoAOMT and CCoAOMT-like promoters, and they are scattered throughout the sorghum genome.

The clusters of lignification-associated transcription factor binding sites were identified in the promoters of two genes: SbCCoAOMT-1 (six binding sites) and SbCCoAOMT-4 (four binding sites), Fig. 3. The clusters are composed of two motifs: (1) MybPlant motif (Place s000167), related to the P-box motif in promoters of phenylpropanoid biosynthesis genes that also regulate lignin biosynthesis (Tamagnone et al. 1998), and (2) ACIIPVPAL2 motif required for vascular-specific expression of phenylalanine ammonia lyase 2 in bean and involved in xylem-localized regulation of genes for lignin biosynthesis enzymes (Patzlaff et al. 2003). The clusters of these motifs are present exclusively in SbCCoAOMT-1 and SbCCoAOMT-4 genes where they occupy similar localization: − 274 to + 248 bp in CCoAOMT-1 and − 247 to + 14 bp in CCoAOMT-4.

Searching sorghum CCoAOMT and CCoAOMT-like promoter regions for AC regulatory elements associated with lignification (Raes et al. 2003; Weng et al. 2008) identified six highly similar or identical motifs. Five of these motifs are near the transcription start sites of the SbCCoAOMT-1 and SbCCoAOMT-4 genes within the clusters of the MybPlant and ACIIPVPAL2 transcription factor-binding sites, Fig. 3.

### Expression of *S. bicolor* CCoAOMT and CCoAOMT-like genes in field-grown plants

**Fig. 4:**
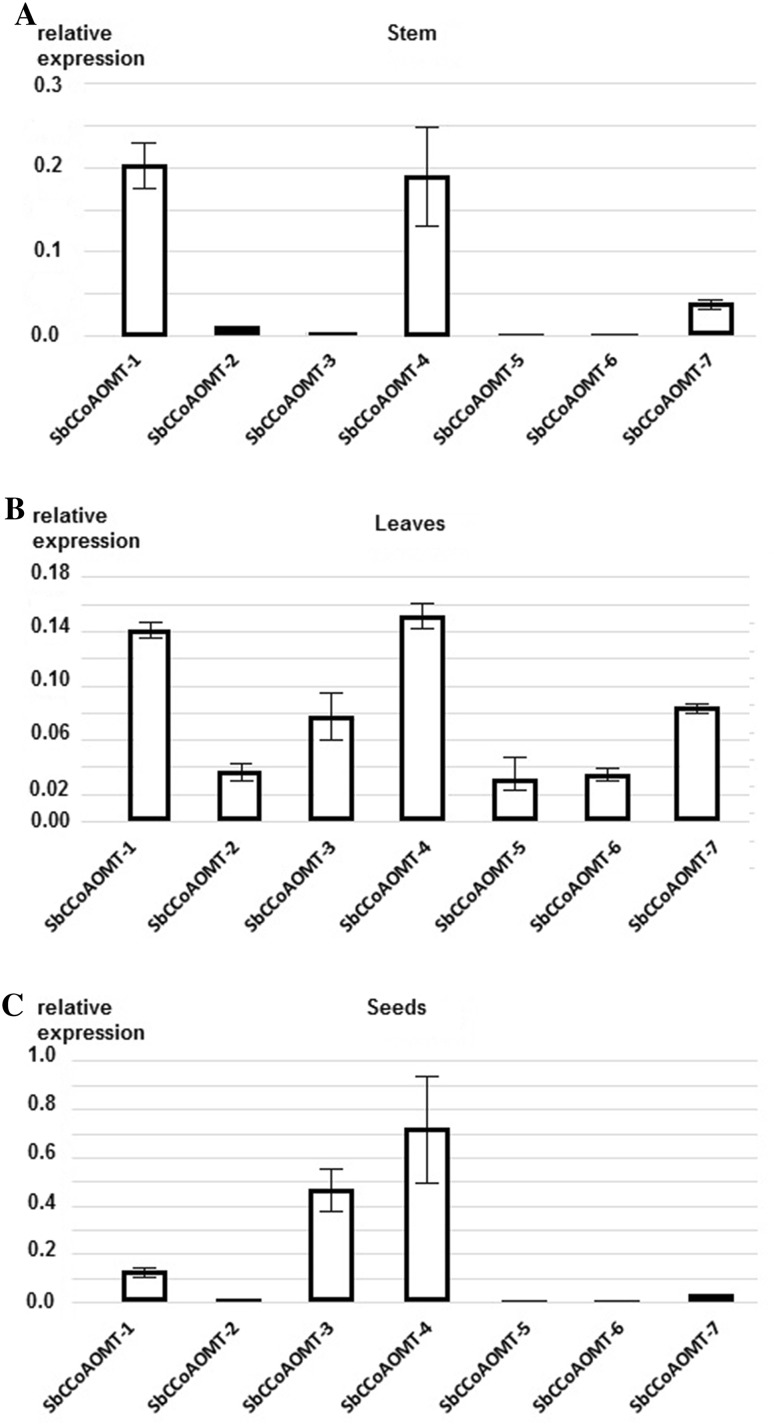
Relative expression of *S. bicolor* CCoAOMT and CCoAOMT-like genes in **a** the inner section of the stem, **b** mature leaves, and **c** immature seeds. The transcript levels were quantified using RT-ddPCR and normalized relative to the expression of the Sb09g023560 gene, which encodes a ubiquitin-conjugating enzyme; values are the mean of two independent RT-ddPCR experiments ± SD

The expression of the sorghum CCoAOMT and CCoAOMT-like genes in the inner section of the stem, mature leaves and immature seeds of field-grown plants was analyzed by RT and digital PCR, Fig. 4. The Sb09g023560 gene encoding a ubiquitin-conjugating enzyme expressed at a constant and high level was used for data normalization between different samples (Johnson et al. 2014).

Two genes, SbCCoAOMT-1 and SbCCoAOMT-4, were expressed at similar, high levels in the stems of mature sorghum plants. The transcripts of these genes were approximately five times less abundant than transcripts of the reference gene Sb09g023560. The expression of the other sorghum CCoAOMT-like genes in the stem was 5–100 times lower than that of SbCCoAOMT-1 and SbCCoAOMT-4. In leaves, the expression of SbCCoAOMT-1 and SbCCoAOMT-4 was still dominant but lower than in the stem, and the expression of the other genes was 3–5 times lower than that of SbCCoAOMT-1 and SbCCoAOMT-4. In immature seeds, SbCCoAOMT-1, SbCCoAOMT-3 and SbCCoAOMT-4 were highly expressed. The highest expression, three times higher than in the stem, was SbCCoAOMT-4. The SbCCoAOMT-1 gene was expressed in seeds at a similar level as in the stem, and the expression of SbCCoAOMT-3 was between SbCCoAOMT-4 and SbCCoAOMT-1.

## Discussion

The sorghum genome contains seven CCoAOMT and CCoAOMT-like genes. One of them, SbCCoAOMT-1, belongs to clade 1a comprising substrate-specific CCoAOMTs (Paterson et al. 2009; Walker et al. 2016). The high expression of SbCCoAOMT-1 gene in stems and leaves, organs of high lignification, is characteristic for this clade, Fig. 4. In this study, SbCCoAOMT-4, one of clade 1c genes, showed high expression similar to SbCCoAOMT-1 in leaves and stems. The promoter regions of both these genes possess clusters of transcription factor-binding sites and AC elements associated with lignification, Fig. 3. These findings were supported by localization of these two proteins in the cytoplasm, and their ability to catalyze caffeoyl-coenzyme A methylation to feruloyl-coenzyme A (Walker et al. 2016), which strongly suggest that SbCCoAOMT-1 and SbCCoAOMT-4 mainly function in monolignol synthesis associated with lignification, Fig. 1 and Table 2.

Apart from the highly expressed SbCCoAOMT-1 and SbCCoAOMT-4 genes, the SbCCoAOMT-2, SbCCoAOMT-3, SbCCoAOMT-5, SbCCoAOMT-6 and SbCCoAOMT-7 genes had activity in mature leaves, but it was 2–7 times lower. High diversity of CCoAOMT-like gene expression in leaves might be associated with the exposure of these organs to a variety of stresses, such as mechanical stress, damage, biological pathogens, high temperature, or intensive light irradiation. In seeds, the expression of the SbCCoAOMT-4 and SbCCoAOMT-3 genes exceeded that of SbCCoAOMT-1, which was expressed at a similar level as in leaves and stem, Fig. 4. The role of these genes in seeds is probably associated with forming protective structures of lignin. The SbCCoAOMT-3 protein, which possesses N-terminal extension and is probably targeted to cytosol and plastids, might be involved in synthesizing some protectants.

The SbCCoAOMT-6 gene encodes the shortest of 44 analyzed proteins, including the cyanobacterial protein. A previous report suggested that the SbCCoAOMT-6 protein is incomplete and lost enzymatic activity due to large deletions and insertion (Walker et al. 2016), Fig. S-1. The unusual structure of the SbCCoAOMT-6 gene and exon–intron boundaries different from the genes from superclade 1 and clade 2, are suggesting that the gene might have arisen as a result of pseudogene activation.

Forty-five highly conserved amino acids were identified in the set of 43 proteins used for the phylogenetic analysis and more than half of them are preserved in all analyzed proteins, Fig. S-1 (100% conserved amino acids are labeled with black dots). The high conservation of amino acids results from interactions preventing their substitution and may be used for protein structural analysis. The role of one-third of the highly conserved amino acids was assigned in previous studies on various plants (Brandt et al. 2015; Ferrer et al. 2005; Hoffmann et al. 2001; Kopycki et al. 2008a, b; Walker et al. 2016). The highly conserved amino acids correspond to SbCCoAOMT-1 Gly-101, Thr-104, Ser-107, Asp-177, Asp-203, and Asn-204, which are involved in SAM or divalent metal ion binding, or Ala-178, Lys-180, and Asp-252 involved in interactions with methyl group acceptors or transmethylation products. Five other highly conserved amino acids correspond to *M. sativa* ACC28973 Glu-85 and Asp-165 involved in SAM and metal binding, *Synechocystis* sp. CCoAOMT-like Lys-3 and Trp-173, and *N. tabacum* ACC49913 Arg-220, participating in interactions with the methyl donor or acceptor (SbCCoAOMT-1 corresponding amino acids, respectively: Glu-99, Asp-179, Lys-35, Trp-207, Arg-242).

Nonetheless, a majority of the highly conserved amino acids, including seventeen 100% conserved amino acids, were not previously identified as essential for CCoAOMT and CCoAOMT-like enzymes. Analysis of the SbCCoAOMT-1 model suggests that Tyr-46, which has 100% conservation, together with a less-conserved Leu-43, located in the close vicinity of N-terminal dimerization domains, might be involved in dimer formation, Fig. S-2. The high conservation of Val-102 and Gly-105, along with Tyr-106 (with 95% conservation), which are located between amino acids directly interacting with SAM, suggests their importance for SAM positioning and orientation. The other group of 100% conserved amino acids includes Phe-175, Leu-193 and Gly-198 together with Gly-197 (with 95% conservation) and seems to be essential for the positioning and orientation of amino acids interacting with SAM or the methyl group acceptor, Fig. S-2.

The phylogenetic analysis suggests that the dedifferentiation of clade 1a from clade 2 is associated with early stages of land plant evolution. As a result, the sorghum proteins from these clades have been separated for approximately 430 MYA (Labeeuw et al. 2015; Renault et al. 2017; Weng and Chapple 2010). Despite such long independent evolution, the structures of SbCCoAOMT-1 and SbCCoAOMT-7 proteins appear to be highly similar, Fig. 2. The most significant differences in the structures of these proteins depend on three regions: (1) Asp-42–Thr-50 from the dimerization region, (2) Ala-154–Asp-164, and (3) Leu-213–Lys-221 in the insertion loop (all coordinates according to SbCCoAOMT-1). The dimerization region Asp-42–Thr-50 contains two highly conserved amino acids preserved probably due to their importance for dimer formation, Figs. S-1 and S-2. The region Ala-154–D-164 contains one highly conserved amino acid that is involved in interactions with the methyl donor. The region of the highest discrepancy between SbCCoAOMT-1 and SbCCoAOMT-7 is located between Leu-213–Lys-221, in the SbCCoAOMT-1 insertion loop, which does not contain any highly conserved amino acids. The strong structural differences in this region associated with sequence remodeling and deletions in the SbCCoAOMT-7 insertion loop, suggest that SbCCoAOMT-7 uses a different methyl acceptor than SbCCoAOMT-1, Fig. S-1. The insertion loop, which is highly variable between clades, shows high conservation within clade 1a and 2, Fig. S-2.

The CCoAOMT and CCoAOMT-like proteins phylogeny together with the specificity of gene structures within the clades, suggest that cyanobacterial genes from clade 2 are ancestors of all CCoAOMT and CCoAOMT-like genes. The presence of the genes from clades 1a and 2 in the *Physcomitrella patens* genome and the identification of clade 1a gene in liverwort *P. appendiculatum* suggest that superclade 1 arose at the very early stage of land plant evolution (Xu et al. 2015). During manuscript preparation, the *Marchantia polymorpha* genome sequence was released (Bowman et al. 2017). This allowed us to identify two liverwort CCoAOMT genes from clade 1a and one CCoAOMT-like gene from clade 2 (not shown). The *M. polymorpha* CCoAOMT-like genes are the most closely related to *P. appendiculatum, Ph. patents*, or S. *moellendorffii* homologs and possess an exon–intron structure characteristic for their clades that reinforce the localization of the origin of superclade 1 on the earliest stage of land plant evolution (Emiliani et al. 2009; Weng and Chapple 2010). The analysis of the *S. moellendorffii* genome identified exclusively clade 1a genes. The lack of *S. moellendorffii* clade 2 homologs might result from genes lost during the evolution of Selaginellaceae, after their dedifferentiation from the other early embryophytes.

In summary, two of seven sorghum CCoAOMT and CCoAOMT-like genes, SbCCoAOMT-1 and SbCCoAOMT-4, revealed high expression in leaves, stem and immature seeds—organs of high lignification activity. Moreover, the clusters of lignin associated transcription factor binding sites are present exclusively in promoters of these two genes; together with a previously reported ability of these enzymes to *O*-methylate caffeoyl-coenzyme A, these results point to the important role of SbCCoAOMT-1 and SbCCoAOMT-4 for lignin synthesis. One of these enzymes, SbCCoAOMT-1, belongs to a clade grouping substrate-specific enzymes known to be involved in lignification, whereas the other, SbCCoAOMT-4, belongs to a clade of substrate-promiscuous CCoAOMT-like enzymes usually associated with species-specific processes, distinct from lignification. The results of this study suggest that enzymatic activity of SbCCoAOMT-4 is associated with lignification, rather than with other pathways; however, more research is required to determine the frequency of such functional reversion in this clade.

The amino acid conservation analysis together with homology modeling allowed us to identify amino acids essential for CCoAOMT and CCoAOMT-like enzymes. The structure of SbCCoAOMT-7, a sorghum protein from the ancestral CCoAOMT-like clade was predicted and compared with SbCCoAOMT-1 from the substrate-specific CCoAOMT clade. The structure comparison allowed us to identify the most structurally divergent regions between these proteins such as the N-terminal α-helix involved in dimerization and the insertion loop involved in methyl acceptor discrimination.

The phylogenetic analysis of CCoAOMT and CCoAOMT-like enzymes categorized these proteins into four clades. The ancestral position of the clade comprising CCoAOMT-like proteins closely related to cyanobacterial enzymes is supported by the gene exon structure specific to this clade.

In conclusion, these findings suggest that genes from both groups, CCoAOMT and CCoAOMT-like, contribute to lignin formation in sorghum and may be the key to improving the quality of plant material for bioenergy applications. Furthermore, the results of the conservation analysis suggest potential targets for site-directed mutagenesis, that may affect CCoAOMT enzyme activity. In addition, the differences in gene structures between the clades provide insight into early stages of the land plant evolution, when the demand for monolignols fixed duplicated CCoAOMT genes. Taken together, the results extend our knowledge of CCoAOMT and CCoAOMT-like genes as an important plant gene family and suggest that substrate-promiscuous CCoAOMT-like enzymes in other plants might also be involved in lignification processes.

## Electronic supplementary material

Below is the link to the electronic supplementary material.

Supplementary material 1 (DOCX 13 KB)

Supplementary material 2 (TIF 272 KB)

Supplementary material 3 (TIF 413 KB)

Supplementary material 4 (TIF 401 KB)

Supplementary material 5 (TIF 372 KB)

Supplementary material 6 (DOCX 13 KB)

B, Anderson R, Olson SN
