# Supplementary material for: Sorghum CCoAOMT and CCoAOMT-like gene evolution, structure, expression and the role of conserved amino acids in protein activity
Source: Mol Genet Genomics. 2018 May 2;293(5):1077–89. doi: 10.1007/s00438-018-1441-6 (PMC6153501; doi:10.1007/s00438-018-1441-6)
Supplement: Supplementary file 1 — Supplementary material 1 (DOCX 13 KB) [file 438_2018_1441_MOESM1_ESM.docx]

**Captions to supplementary figures**

**Supplementary Fig. S-1**

**Fig. S-1** Sequence alignment of sorghum CCoAOMT, sorghum CCoAOMT-like enzymes and homologous proteins. The aligned proteins were organized into clades: 1a – true CCoAOMTs; 1c – CCoAOMT-like enzymes from grasses; and 2 – CCoAOMT-like enzymes closely related to Cyanobacteria; clade 1b is not presented. The presented proteins are: *S. bicolor* - SbCCoAOMT-1 to SbCCoAOMT-7; *A. thaliana* – AT4G34050 and AT3G62000.1; *M. sativa* - AAC28973; *N. tabacum* - AAC49913.1; *M. truncatula* – Medtr4g085590 and Medtr2g070410; *Z. mays* - CAB45149.1, AFW65160, DAA40360, and XP_008662866; *O. sativa* - AAT68023 and BAG98414; *P. patens* - Pp3c4_20870V1.1; *C. reinhardtii* – XP_001693484; and *Synechocystis* sp.- WP_010873795.1.

Amino acids of conservation higher than 80% are highlighted in black for fully conserved amino acids or in gray for conservative substitutions. The highly conserved amino acids shown in the top and bottom lanes (conserved aa) are of above 95% conservancy within set of 43 proteins used for the phylogenetic study (SbCCoAOMT-6 was excluded from the analysis); amino acids of 100% conservancy, i.e. conserved in all 43 proteins, are marked with black circles.

Amino acids involved in interactions with a methyl group donor are marked with arrowheads, amino acids involved in interactions with metal ions are marked with exclamation mark, and amino acids involved in interactions with a methyl group acceptor or transmethylation product are marked with asterisks; amino acids where these interactions have been identified are framed in red. Amino acids involved in dimer formation are marked with forward slashes, variable loop is marked with empty rectangle, and the regions of the highest discrepancy between 3-D structure of SbCCoAOMT-1 and SbCCoAOMT-7 are marked with dotted line.

Amino acids corresponding to the 3’ end of exons are underlined in red.

**Supplementary Fig. S-2**

**Fig. S-2** Conserved residues in the CCoAOMT peptides mapped to the structure of SbCCoAOMT-1 (PBD: 5KVA). The dark blue color indicates high conservation; red indicates high variance; divalent metal ion - Ca2+, is shown in green. Highly conserved amino acids of unknown functions/interactions are labeled.

**Supplementary Tab. S-1**

**Tab. S-1** Long conserved motifs located in the transcription start site upstream region of SbCCoAOMT and SbCCoAOMT-like genes. The longest motif within a class is bolded.
