# Supplementary figures and images for: Sorghum CCoAOMT and CCoAOMT-like gene evolution, structure, expression and the role of conserved amino acids in protein activity
Source: Mol Genet Genomics. 2018 May 2;293(5):1077–89. doi: 10.1007/s00438-018-1441-6 (PMC6153501; doi:10.1007/s00438-018-1441-6)

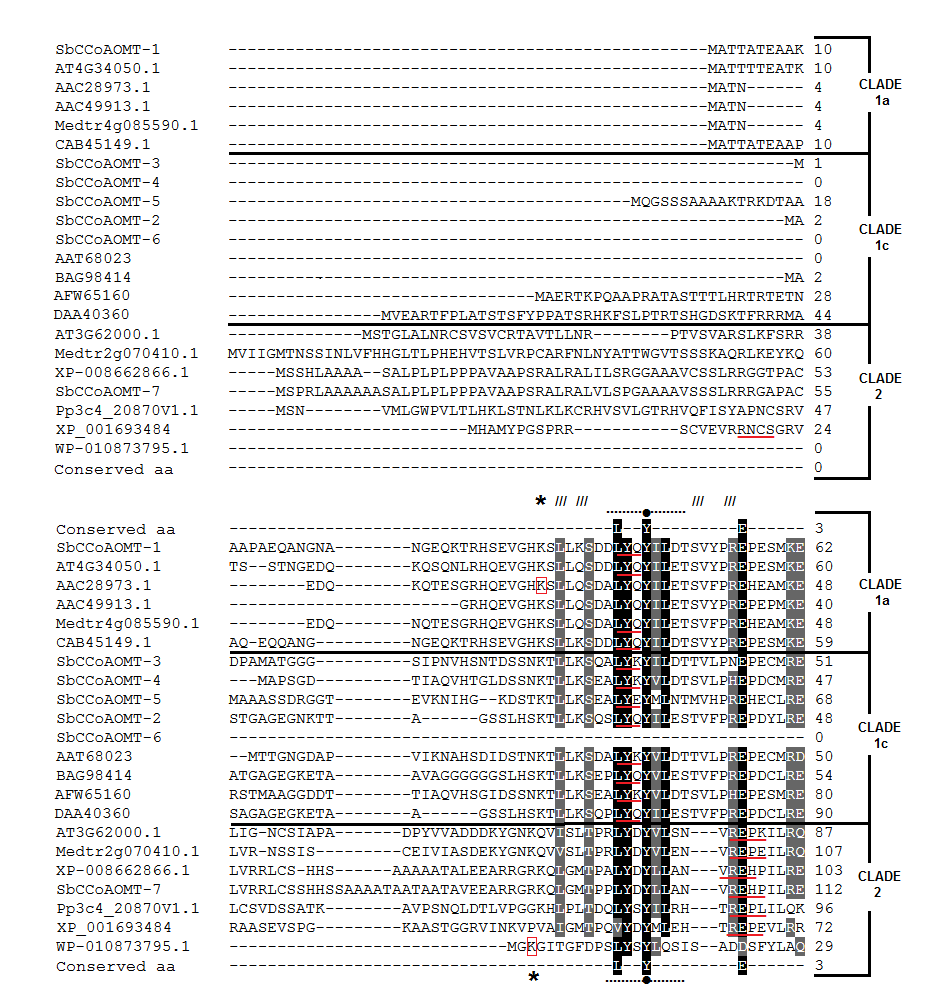

Supplement: Supplementary file 2 — Supplementary material 2 (TIF 272 KB) [file 438_2018_1441_MOESM2_ESM.tif]

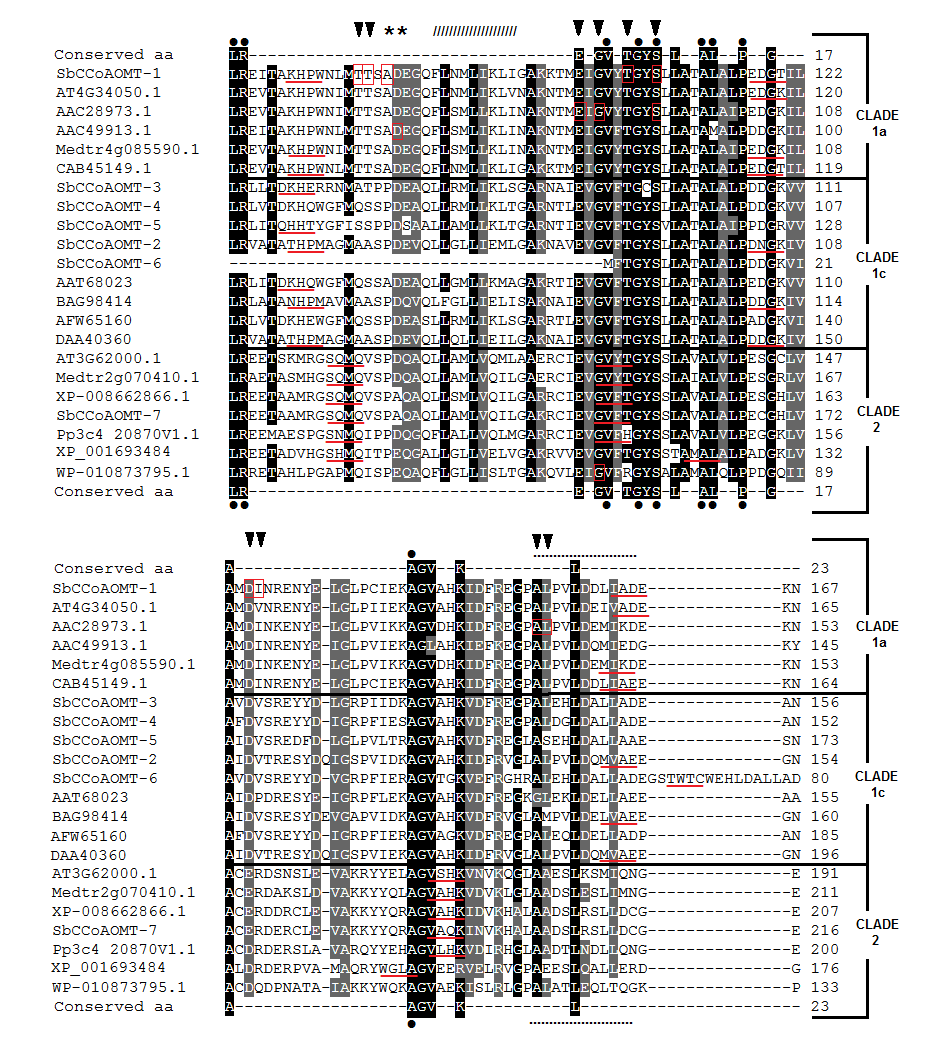

Supplement: Supplementary file 3 — Supplementary material 3 (TIF 413 KB) [file 438_2018_1441_MOESM3_ESM.tif]

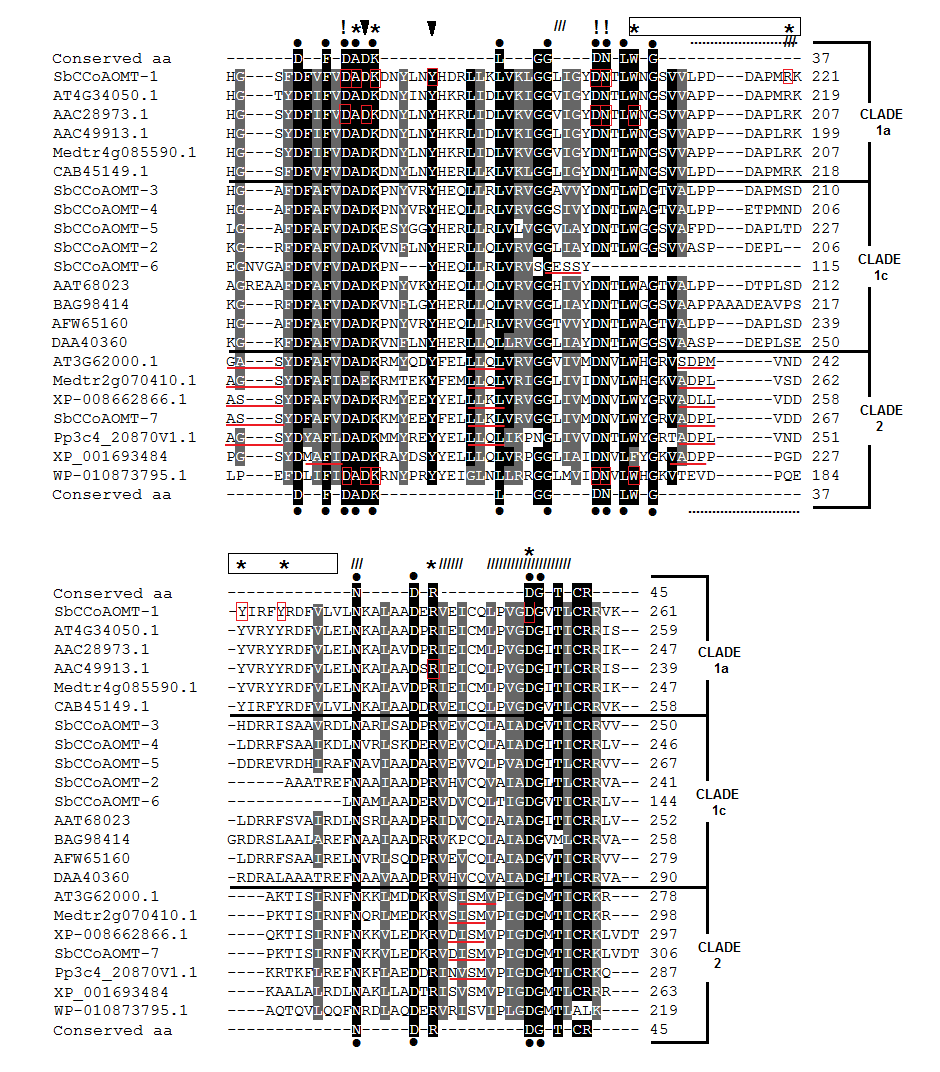

Supplement: Supplementary file 4 — Supplementary material 4 (TIF 401 KB) [file 438_2018_1441_MOESM4_ESM.tif]

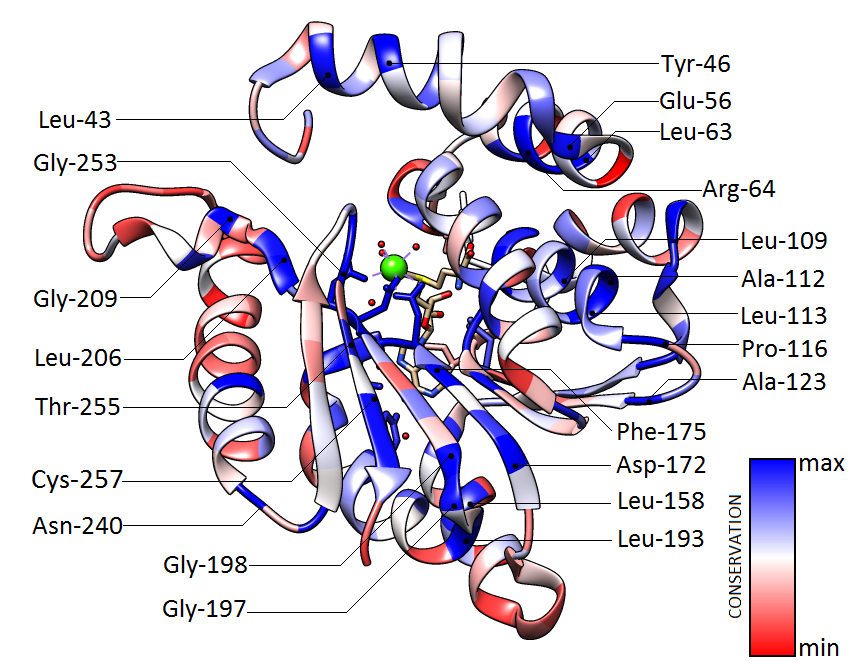

Supplement: Supplementary file 5 — Supplementary material 5 (TIF 372 KB) [file 438_2018_1441_MOESM5_ESM.tif]
