# Supplementary material for: Sorghum CCoAOMT and CCoAOMT-like gene evolution, structure, expression and the role of conserved amino acids in protein activity
Source: Mol Genet Genomics. 2018 May 2;293(5):1077–89. doi: 10.1007/s00438-018-1441-6 (PMC6153501; doi:10.1007/s00438-018-1441-6)
Supplement: Supplementary file 6 — Supplementary material 6 (DOCX 13 KB) [file 438_2018_1441_MOESM6_ESM.docx]

| Gene/protein id in this study  / Phytozome locus id | Localization regard TSS,  Motif length, motif identity to the longest motif within a class | | | |
| --- | --- | --- | --- | --- |
|  | Motif A | Motif B | Motif C | Motif D |
|  |  |  |  |  |
| SbCCoAOMT-1  / Sobic.010G052200.1 | x | -2781.. -2584  198 bp, 84% | x | x |
|  |  |  |  |  |
| SbCCoAOMT-2  / Sobic.002G242300.1 | -1956..-1690  267 bp, 78% | x | x | x |
|  |  |  |  |  |
| SbCCoAOMT-3  Sobic.007G218700.1 | **-1850..-1582**  **269 bp, 100%** | -312.. -114  199 bp, 79% | x | **-665..-516**  **150 bp, 100%** |
|  |  |  |  |  |
| SbCCoAOMT-4  / Sobic.007G218800.1 | -422..-385  38 bp, 79% | -2194.. -1995  200 bp, 75% | x | x |
|  |  |  |  |  |
| SbCCoAOMT-5  / Sobic.007G218500.1 | x | -3000..-2833  167 bp, 75% | x | x |
|  |  |  |  |  |
| SbCCoAOMT-6  / Sobic.007G217200.1 | x | **-1352..-1141**  **212 bp, 100%** | **-1060..-700**  **361 bp, 100%** | x |
|  |  |  |  |  |
| SbCCoAOMT-7  / Sobic.007G043200.1 | x | x | -474..-115  360 bp, 97% | -966..-870  97 bp, 74%  -840..-721  84 bp, 77% |
